# Dynamic Changes in Antioxidant Activity and Ethanol Adsorption Capacity of Sugarcane Vinegar During In Vitro Digestion: Insights into the Prevention of Alcoholic Liver Disease

**DOI:** 10.3390/foods15152759

**Published:** 2026-08-06

**Authors:** Feifei Wu, Fengjin Zheng, Bo Lin, Hao Cheng, Yuan Tan, Yuxia Yang, Krishan K. Verma, Ganlin Chen

**Affiliations:** 1 Guangxi South Subtropical Agricultural Research Institute, Guangxi Academy of Agricultural Sciences, Chongzuo 532400, China; wufeifei@gxaas.net; 2 Guangxi Key Laboratory of Green Processing of Sugar Resources, College of Biological and Chemical Engineering, Guangxi University of Science and Technology, Liuzhou 545006, China; iamchenghao@126.com; 3 Institute of Agro-Products Processing Science and Technology, Guangxi Academy of Agricultural Sciences, Nanning 530007, China; linbo@gxaas.net (B.L.); yangyuxia@gxaas.net (Y.Y.); 4 College of Agriculture, Guangxi University, Nanning 530004, China; 18373438400@163.com; 5 Sugarcane Research Institute, Guangxi Academy of Agricultural Sciences, Nanning 530007, China; drvermakishan@gmail.com; 6 Guangxi Subtropical Crops Research Institute, Guangxi Academy of Agricultural Sciences, Nanning 530001, China; 7 Guangxi Key Laboratory of Quality and Safety Control for Subtropical Fruits, Nanning 530001, China; 8 Key Laboratory of Quality and Safety Control for Subtropical Fruit and Vegetable, Ministry of Agriculture and Rural Affairs, Nanning 530001, China

**Keywords:** sugarcane vinegar, active ingredients, bioaccessibility, alcoholic liver disorder, action mechanism

## Abstract

This study used an in vitro simulated digestion model combined with network pharmacology to investigate the bioaccessibility, dynamic changes in antioxidant capacity, and potential molecular mechanisms by which active components of sugarcane vinegar mitigate alcoholic liver disease (ALD). The results showed that the bioaccessibility of total phenols, total flavonoids, and reducing sugars in sugarcane vinegar exceeded 90% during gastric digestion. However, after combined gastrointestinal digestion, the bioaccessibility of total flavonoids was reduced significantly (39.39%). Among the phenilic phenolic monomers, ferulic acid had the highest gastric bioaccessibility index (419.10%), while luteolin exhibited the strongest stability throughout digestion process. The 2,2-Diphenyl-1-picrylhydrazyl (DPPH) free radical scavenging and total reducing capacity gradually decreased, whereas the 2,2′-azinobis-3-ethylbenzothiazoline-6-sulfonic acid (ABTS) reached its peak during gastrointestinal digestion. Correlation analysis showed that antioxidant capacity was closely correlated with phenolic compounds. Alcohol detoxification experiments revealed the ethanol retention rate of sugarcane vinegar during gastric digestion was significantly higher (14%) than undigested sample. Network pharmacology analysis identified caffeic, ferulic, protocatechuic, vanillic, and gentisic acid in sugarcane vinegar, with MAOB, PTGS1, and PTGS2 targets. These molecular docking findings reveal the strong binding affinity between polyphenols and their core targets. This research establishes a robust theoretical framework for harnessing sugarcane vinegar bioactive properties as a functional food strategy to mitigate alcoholic liver disease.

## 1. Introduction

Chinese baijiu, also known as Chinese national liquor, has a long history and is not only a traditional beverage, but also carries rich cultural connotations and social functions, becoming an important medium for interpersonal communication [1]. However, long-duration excessive drinking may lead to varying degrees of liver cell damage, which in turn can induce abnormal liver functions. The liver is the core organ for ethanol metabolism and therefore is most susceptible to damage from alcohol and its metabolites [2]. Alcoholic liver disease (ALD) is the second most prevalent liver disease globally, following viral hepatitis, and represents a major cause of mortality worldwide [3].

Currently, intervention strategies for ALD mainly focus on different pathways, such as accelerating the clearance of ethanol and its toxic metabolites, i.e., acetaldehyde; enhancing the body’s ability to scavenge free radicals and reducing oxidative stress damage; and inhibiting inflammatory responses and blocking the amplification effect of the liver inflammatory cascade [4,5,6]. In recent years, functional foods derived from the natural products have garnered significant interest for preventing and treating ALD, owing to their multi-component synergistic effects and multi-target mechanisms [7,8,9]. Vinegar, a fermented condiment, is rich in various bioactive components, i.e., organic acids, polyphenols, and flavonoids, and has been shown to have multiple physiological functions, including antioxidation, anti-inflammation, and lipid reduction [10,11,12]. However, sugarcane vinegar, fermented and brewed from sugarcane, has gradually attracted researchers’ attention for its unique flavor and rich phenolic composition. Previous studies found that sugarcane vinegar polyphenols not only exhibit significant 2,2-Diphenyl-1-picrylhydrazyl (DPPH) and 2,2′-azinobis-3-ethylbenzothiazoline-6-sulfonic acid (ABTS) free radical scavenging activity but also reduce oxidative stress in mice and show potential anti-inflammatory activity [1]. Based on the research findings, sugarcane vinegar may have the potential to combat alcoholic liver damage.

However, the active ingredients in functional foods are influenced by pH changes, digestive enzyme activities, and gut microbiota, during passage through the human gastrointestinal environment, resulting in varying degrees of chemical transformation or degradation, thereby reducing their bioaccessibility and physiological activity [13,14,15]. Currently, no systematic studies have reported on the stability, release patterns, and bioaccessibility of active ingredients in sugarcane vinegar during digestion. In vitro simulated digestion models have been widely applied to assess the dynamic changes of active ingredients, such as polyphenols and flavonoids, in food matrices in the oral cavity, stomach, and small intestine, and the results can provide significant references for in vivo bioaccessibility studies [16,17,18]. In addition, network pharmacology, as a systems biology method, can predict the potential molecular mechanisms by constructing “compound–target–pathway” networks to predict the synergistic effects of multiple components in natural products on disease targets, and has been widely used in the traditional Chinese medicine and functional food research in recent years [19,20,21].

The present study employed an in vitro simulated digestion model to systematically evaluate the changes in the content and bioaccessibility of total phenols, total flavonoids, reducing sugars, and major polyphenol monomers (ferulic acid, caffeic acid, protocatechuic acid, vanillic acid, gentisic acid, etc.) of sugarcane vinegar during the oral, gastric, and small intestinal digestion stages. Simultaneously, the DPPH and ABTS free radical scavenging capacities and the reducing capacity of samples at different digestion stages were measured to reveal the impact of digestion process on the antioxidant activity of sugarcane vinegar. Based on this, network pharmacology approaches were used to screen key active ingredients in sugarcane vinegar for anti-alcoholic liver disease, predict their targets and related signaling pathways, and conduct preliminary verification through molecular docking. This study aims to assess the bioaccessibility of bioactive compounds in sugarcane vinegar during digestion and to elucidate the molecular mechanisms by which its polyphenolic components mitigate alcoholic liver injury through multi-target synergism. The research findings provide a scientific rationale for developing sugarcane vinegar as a functional food for preventing and managing alcoholic liver disease.

## 2. Materials and Methods

### 2.1. Chemicals and Reagents

Rutin, gallic acid, anhydrous glucose, and Trolox were analytical-grade standards purchased from the Shanghai Yuanye Biotechnology Co., Ltd., (Shanghai, China). Luteolin, chlorogenic acid, syringic acid, ferulic acid, vanillic acid, p-coumaric acid, protocatechuic acid, gentisic acid, p-hydroxybenzoic acid, salicylic acid, and caffeic acid standards were HPLC-grade standards purchased from the Shanghai Yuanye Biotechnology Co., Ltd., (Shanghai, China). HPLC-MS grade acetonitrile and formic acid were provided by the Fisher Scientific (Hampton, NH, USA). 1,1-diphenyl-2-trinitrophenylhydrazine (DPPH) and 2,2-azidobis- (3-Ethylbenzodihydrothiazoline 6-sulfonic acid) diammonium salt (ABTS), sodium acetate, trypsin, pepsin, and Folin–Ciocalteu reagent were analytical grade and purchased from the Solarbio, Beijing, China. Anhydrous ethanol and acetone were chromatographic grade, and methanol, ethyl acetate, glacial acetic acid, and other chemicals were purchased from the Chengdu Kelong Chemical Co., Ltd., Chengdu, China. Brewer’s yeast and lactic acid bacteria (LB) were provided by the Angel Yeast Co., Ltd., Yichang, China and Shanxi Dingfeng Brewing Technology Co., Ltd., Xianyang, China.

### 2.2. Preparation of Samples

Sugarcane vinegar was prepared according to our previous method [1]. Sugarcane juice was adjusted to 20° Brix by adding sugar, inoculated with 10 g/L of fruit wine yeast (Yichang Anji Yeast Co., Ltd., Yichang, China), and fermented at 27 °C (18 days). After the fermentation, the liquid was diluted twice with sterile water, 5 g/L of LB acetic acid bacteria was added, and the mixture was fermented at 25 °C for 20 days. At final stage, the fermentation liquid was filtered and sterilized at 80 °C (30 min) to obtain sugarcane vinegar (pH 3.02 ± 0.05).

### 2.3. Analysis of Total Phenolic Content, Total Flavonoids, and Reducing Sugars

Total phenol content (TPC) was quantified via the Folin–Ciocalteu colorimetric method according to the procedure of Wu et al. [1] and Özdemir et al. [10]. A 150 µL aliquot of the diluted sample was combined with 750 µL of 10% Folin–Ciocalteu reagent, incubated for 5 min, and subsequently mixed with 600 µL of 7.5% (*w*/*v*) Na_2_CO_3_. The resulting solution was protected from the light for 90 min. The optical density was measured at 760 nm, and TPC was expressed as µg/mL of gallic acid equivalents.

Total flavonoid content (TFC) was determined according to the procedure of Bai et al. [11]. An appropriately diluted sample was combined with 0.7 mL of 5% NaNO_2_, incubated for 6 min, and subsequently treated with 0.7 mL of 10% Al(NO_3_)3. The mixture was allowed to stand for 6 min, and then 2 mL of 4% NaOH was added, and the volume was maintained to 10 mL with ethanol (60%). Following 10 min incubation at room temperature, absorbance was measured at 510 nm. Total flavonoid content (TFC) was subsequently expressed in rutin equivalents (µg/mL).

Reducing sugar content (RSC) was quantified using a modified DNS (3,5-dinitrosalicylic acid) method based on the protocol described by the Jakubczyk et al. [22]. The diluted sample (1 mL) was combined with 2 mL of DNS reagent and vigorously shaken to ensure thorough mixing. The mixture was incubated in boiling water (5 min), cooled to room temperature (20 to 25 °C), and diluted to a final volume of 15 mL with distilled water. Reducing sugar content was determined spectrophotometrically at 540 nm and expressed relative to a glucose standard (mg/mL).

### 2.4. In Vitro Gastrointestinal Digestion Procedure

The experiment was conducted according to Li et al. [12,13], with slight modifications. Simulated salivary fluid (SSF), simulated gastric fluid (SGF), and simulated intestinal fluid (SIF) were prepared using the formulations presented in Table 1 [13]. The simulated in-vitro digestion working solutions were diluted to a final volume of 500 mL with distilled water. A 1 mol/L sodium hydroxide solution or a 6 mol/L hydrochloric acid solution was prepared, and the pH was adjusted to 3.0 and 7.0, respectively. The simulated digestion solutions were stored at −20 °C for up to one year or at 2–5 °C for about one month.

#### 2.4.1. Simulated Oral, Gastric, and Intestinal Digestion

Mix 10 mL of sugarcane vinegar with 10 mL of SSF until homogeneous, then add 0.3 mol/L CaCl(H_2_O)_2_ to achieve an SSF concentration of 1.5 mmol/L. Incubate at 37 °C (100 rpm) for 2 min on a shaker. After digestion, remove the centrifuge tube, immediately stop the reaction on ice, and centrifuge at 4000 rpm (10 min). Store the supernatant (S_1_) at −20 °C. Dilute the sugarcane vinegar with distilled water to the same volume as the digested sample (oral, gastric, and intestinal digestive fluids) as a blank control (CK). Thaw the sample prior to testing and then filter it through a 0.22 µm microporous membrane.

A 10 mL quantity of the orally digested sample solution was added to 10 mL of simulated gastric juice. The pH was adjusted to 3.0 by adding 0.6 mol/L hydrochloric acid (HCl). CaCl(H_2_O)_2_ was added to achieve an SGF concentration of 0.15 mmol/L, and pepsin was added to achieve an SGF concentration of 120 U/mL. The mixture was incubated at 37 °C (100 rpm) for 2 h on a shaker. After digestion, the centrifuge tube was removed, and the reaction was immediately stopped by placing it on ice. The mixture was subsequently centrifuged at 4000 rpm for 10 min, after which the supernatant (S_2_) was stored at −20 °C. The orally digested sample was adjusted to pH 3.0 using HCl as a gastric acid control (CKG). Samples were thawed and filtered through a 0.22 µm microporous membrane before testing.

A 10 mL quantity of the gastric-digested sample solution was added to 10 mL of simulated intestinal fluid. The pH was adjusted to 7.0 using 1 mol/L NaOH. CaCl(H_2_O)_2_ was added to achieve a SIF concentration of 0.15 mmol/L. 4 mL of trypsin–bile mixture (1.9 g porcine bile salts and 0.3 g trypsin dissolved in 60 mL of 0.1 mol/L NaHCO_3_ buffer) was added. The mixture was incubated at 37 °C for 2 h at 100 rpm on a shaker. After digestion, the centrifuge tube was removed, and the reaction was immediately stopped by placing it on ice. The mixture was then centrifuged at 4000 rpm for 10 min, and the supernatant (S_3_) was stored at −20 °C. Prior to testing, samples were thawed and filtered through a 0.22 µm microporous membrane.

#### 2.4.2. Bioaccessibility of Active Ingredients

Biological accessibility (BI) is the content ratio of active substances in gastric and intestinal digestive fluids to the content of active substances in raw sugarcane vinegar before digestion.

$$BI\;\left(\%\right)=\left(\frac{C_{MI}}{C_{IN}}\right)\times 100$$

In the formula, *BI* represents the bioaccessibility of the active substance, *C_IN_* refers to the content of active substances before sugarcane vinegar digestion, and *C_MI_* is the active substances content in the supernatant after centrifugation of gastric and intestinal digestive fluids.

### 2.5. Analysis of DPPH, ABTS Radical Scavenging Capacity, and Total Reducing Power

The DPPH radical scavenging capacity was determined following the method of Li et al. [12] with minor modifications. In a 96-well plate, 100 µL of DPPH methanol solution (100 µg/mL) was mixed with 100 µL of sample solutions at various concentrations. The mixed reaction solution was incubated in the dark for 6 min, then absorbance was measured at 734 nm using a BioTek Epoch microplate reader (BioTek, Shoreline, WA, USA). The mixture of 100 µL DPPH and 100 µL of the test solution was used as a control, and radical scavenging capacity was quantified as Trolox equivalent antioxidant capacity (TEAC, mgTE/100 mL).

The ABTS radical scavenging capacity was assessed using the method described by Boasiako et al. [23]. In brief, 150 µL of ABTS working solution was combined with 50 µL of sample aliquots of varying concentrations in a 96-well microplate. The reaction solution was incubated in the dark for 6 min, then absorbance was measured at 734 nm using a BioTek Epoch microplate reader (BioTek, USA). A standard curve was prepared by measuring the absorbance decrease of ABTS^•+^ across a range of Trolox concentrations. Appropriate control sample measurements were performed, and the values were monitored. The results are quantified as Trolox equivalent antioxidant capacity (TEAC) and expressed in mg TE/100 mL.

Total reducing power (TRP) was determined using the method of Zhang et al. [24]. Briefly, 1 mL of the digested sample was combined with 2.5 mL of phosphate buffer (pH 6.6) and 2.5 mL of potassium ferricyanide (1 g/100 mL), then incubated at 50 °C for 20 min. Following incubation, 2.5 mL of 10% (*m*/*v*) trichloroacetic acid was added to the mixture, and the resulting solution was centrifuged at 5000× *g* for 15 min. Supernatant (2.5 mL) was incubated with 2.5 mL of deionized water and 0.5 mL of 0.1% (*m*/*v*) ferric chloride solution for 10 min. Absorbance at 700 nm was measured using a BioTek Epoch microplate reader (BioTek, USA), with deionized water serving as a control. Total reducing power was quantified as Trolox equivalent antioxidant capacity (TEAC, mg TE/100 mL).

### 2.6. Determination of Ethanol Adsorption Capacity

Ethanol retention rate (ERR) refers to the process by which alcohol-degrading substances bind ethanol, thereby reducing the amount of free ethanol and inhibiting or delaying its absorption in the body. The determination of ethanol retention rate is based on the procedure of Yang et al. [25] with minor modifications. Preparation of test solution of as 0.2 mL of anhydrous ethanol and 0.25 mL of the sample solution to a 10 mL stoppered volumetric flask, shake well, let stand for 1 h, then add 0.2 mL of anhydrous n-propanol, and finally dilute to the mark with acetone to determine the ethanol content in the solution.

$$ERR=\left(C_{0}-C\right)/C_{0}\times 100\%$$

In the formula, *ERR* is the ethanol retention rate, *C*_0_ and *C* is the initial ethanol concentration and free ethanol concentration after incubation.

Ethanol content was determined by gas chromatography in accordance with the method described in Appendix IX M of the *Chinese Pharmacopoeia* (2010 Edition, Volume I) [26]. An internal standard method employed n-propanol as the reference standard was used for quantification. Samples were analyzed using a Gas chromatograph GC-2030 (Shimadzu, Shanghai, China). The chromatographic conditions were as follows: temperature program (initial temperature 50 °C, hold for 7 min, increase to 110 °C at 10 °C/min, hold for 3 min), carrier gas (N_2_: 3.0 mL/min), H_2_ (47.0 mL/min), air flow rate (400 mL/min), detector temperature (220 °C), injection port temperature (200 °C), and split ratio (100:1).

### 2.7. Phenolic Components Analyses

The polyphenolic compounds in the samples of sugarcane vinegar were analyzed using Waters EVEOTQ-S ultra-high-pressure liquid chromatography equipped with an electrospray ionization source (Waters Technology Shanghai Co., Ltd., Shanghai, China). The chromatographic conditions employed for phenolic compounds were as follows: Column: ACQUITYUPLC@HSST3 (2.1 mm × 100 mm, 1.7 µm, Waters, Milford, MA, USA); Mobile phase A: 0.1% formic acid and ultrapure water; Mobile phase B: 100% acetonitrile solution; Flow rate: 0.25 mL/min; Gradient elution program: 0–0.80 min, 90% A; 0.8–4.0 min, 90–80% A; 4.0–6.5 min, 80% A; 6.5–7.0 min, 80–55% A; 7.0–13.0 min, 55% A; 13.0–13.5 min, 55–90% A; 13.5–14.0 min, 90% A; Column temperature (35 °C), injection volume (2 µL). Mass spectrometry was conducted using electrospray ionization source (ES+, ES−), in multiple reaction monitoring (MRM) scan mode, capillary voltage (3.0 kV), ionization source temperature (350 °C), desolvation gas (N_2_), desolvation gas flow rate (700 L/h), cone gas flow rate (150 L/h), collision gas (Ar) flow rate (0.10 mL/min). Phenolic compounds were identified by comparing their retention times with those of corresponding standards, and quantitative analysis was conducted using the external standard method.

### 2.8. Network Pharmacology

#### 2.8.1. Screening Candidate Compounds and Identification of Potential Targets

Targets of sugarcane acetic acid polyphenols were retrieved from the TCMSP database (https://www.tcmsp-e.com/tcmsp.php; accessed on 21 January 2026). The obtained target information was normalized using the UniProt database (https://www.uniprot.org/; accessed on 21 January 2026), and the species was set to Homo sapiens.

Using “Alleviated alcoholic liver injury” as the keyword, targets related to alcoholic liver injury were searched in the GeneCards database (https://www.genecards.org/; accessed on 21 January 2026), OMIM database (https://www.omim.org/; accessed on 21 January 2026), PharmGKB database (https://www.pharmgkb.org/; accessed on 21 January 2026), TTD database (https://db.idrblab.net/ttd/; accessed on 21 January 2026), and DrugBank database (https://go.drugbank.com/; accessed on 21 January 2026). Targets extracted from the five databases were subsequently merged into a unified dataset.

The intersection of the target sites of sugarcane vinegar polyphenols and the targets related to alcoholic liver injury was obtained using the VennDiagram package in R. A network diagram of “sugarcane vinegar polyphenol compounds—targets of alcoholic liver injury” was constructed using Cytoscape 3.7.0 software, showing the intersection targets and their corresponding sugarcane vinegar polyphenol compounds. Network analysis was conducted by examining node degree, where higher degree values signify more important position in the network [1,19].

#### 2.8.2. Pathway Enrichment Analysis

Gene Ontology (GO) is an international standard system for gene function classification. The intersection targets obtained in 2.8.1 were subjected to GO functional enrichment analysis using the KOBAS online analysis platform (http://bioinfo.org/kobas). The top 10 significantly associated items (*p* < 0.05) across biological progress (BP), cellular components (CC), and molecular functions (MF) were selected for visualization and further analysis. KEGG pathway analysis was subsequently performed to elucidate the biological pathways and molecular networks associated with the identified targets, enabling systematic exploration of protein–protein interactions and metabolic reactions [15]. KEGG signaling pathway analysis was performed using the KOBAS online analysis platform (http://bioinfo.org/kobas). The top 10 most significant pathways (*p* < 0.05) associated with alcoholic liver injury were selected for visualization.

#### 2.8.3. Mapping Protein–Protein Interaction and Screening Core Regulatory Targets

Using the STRING database (https://cn.string-db.org/; accessed on 22 January 2026), a PPI network was constructed for the 30 GO functional entries and 10 KEGG signaling pathway targets. Species taxonomy was set to *Homo sapiens*, with all other parameters retained at their default values. The target node data were subsequently exported and visualized as a network diagram using Cytoscape 3.7.0. Targets were selected based on six topological parameters, such as Betweenness, Closeness, Degree, Eigenvector, LAC, and Network, each exceeding the median value. The same method was used to select the targets further. The final results are the core targets of sugarcane vinegar polyphenols in the treatment of alcoholic liver injury.

#### 2.8.4. Molecular Docking Analysis

Molecular docking studies were conducted using the methodology described in the previous work [20]. Briefly, the two-dimensional (2D) structures of sugarcane vinegar polyphenols (ferulic acid, caffeic acid, protocatechuic acid, vanillic acid, and gentisic acid) were retrieved from the PubChem database (https://pubchem.ncbi.nlm.nih.gov/; accessed on 25 January 2026), and their 3D structures were generated using ChemDraw 19.0 software. Protein conformations of the core targets (MAOA, PRKCA, PTGS2, SLC6A3, MAOB, PRKCB, TNF) were obtained from the protein database (PDB, https://www.rcsb.org/; accessed 25 January 2026). Molecular docking simulations between the core targets and polyphenols were conducted using AutoDockTools 1.5.6 to identify the lowest binding energy identified for each molecular pair. The docking results were visualized and analyzed using PyMOL 2.3.2.

### 2.9. Data Analysis

All treatments were performed in triplicate, and experimental data are expressed as mean ± standard deviation (SD). Orthogonal partial least squares discriminant analysis (OPLS-DA) was conducted using SIMCA 14.1 software (Umetrics, Umeå, Sweden). Analysis of variance (ANOVA) was performed using Origin 2021 (OriginLab, Northampton, MA, USA) to identify statistically significant differences (*p* < 0.05).

## 3. Results and Discussion

### 3.1. Dynamic Changes in Total Phenols, Flavonoids, and Reducing Sugars During In Vitro Simulation of Digestive Process

The changes in active ingredients of sugarcane vinegar during simulated digestion are shown in Table 2. After gastric digestion, the total phenol content of sugarcane vinegar reduced significantly. There was no significant difference in the total phenol content of gastric digestive juice, intestinal digestive juice, and gastric acid control solution. The bioaccessibility of gastric and gastrointestinal digestion was high, exceeding 90% in both cases. These results indicate that the overall phenolic pool comprising phenolic acids, such as chlorogenic acid, caffeic acid, ferulic acid, and protocatechuic acid, remains largely stable throughout digestion. Studies have shown that these polyphenols are relatively stable under acidic conditions, and their simple aromatic ring structures confer resistance to degradation [1,27]. In the gastric phase, pepsin plays a dual role in modulating phenolic compound stability. On one hand, pepsin-mediated hydrolysis of ester-linked phenolic acid–polysaccharide complexes may facilitate the release of bound phenolic acids, contributing to the high bioaccessibility of total phenolics observed during gastric digestion. On the other hand, the acidic gastric environment (pH 3.0) itself favors the structural integrity of these phenolic acids. This combined effect explains why the total phenolic content remains relatively stable during gastric digestion despite the presence of proteolytic enzymes [1,27].

Upon entering the intestinal digestion stage, the total flavonoid content of sugarcane vinegar was significantly downregulated. The bioaccessibility of flavonoids in the gastric digestion stage was 97.85 ± 2.69%, while it dropped sharply to 39.68% after gastrointestinal digestion. This marked degradation can be attributed to the structural ability of flavonoids. Flavonoids possess a diphenylpropane (C6-C3-C6) skeleton containing a heterocyclic C-ring that is susceptible to cleavage and oxidative degradation under the neutral pH conditions (pH 7.0) of the intestinal environment, consistent with the results of Wu et al. [28]. As for reducing sugars, bioaccessibility during gastric digestion was high (99.03 ± 1.4%); it dropped to 84.88 ± 1.01% after gastrointestinal digestion. It is worth noting that the reducing sugar content was the lowest in the gastric acid control group (1.43 ± 0.02 mg/mL), while the reducing sugar content in the gastric digestive fluid was as high (1.75 ± 0.07 mg/mL). This phenomenon may be due to pepsin action; some polysaccharides are degraded into reducing sugars by pepsin catalysis [29].

### 3.2. Impact of Antioxidant Capacity of Sugarcane Vinegar During In Vitro Digestion

#### 3.2.1. DPPH Scavenging Capacity

**Figure 1:**
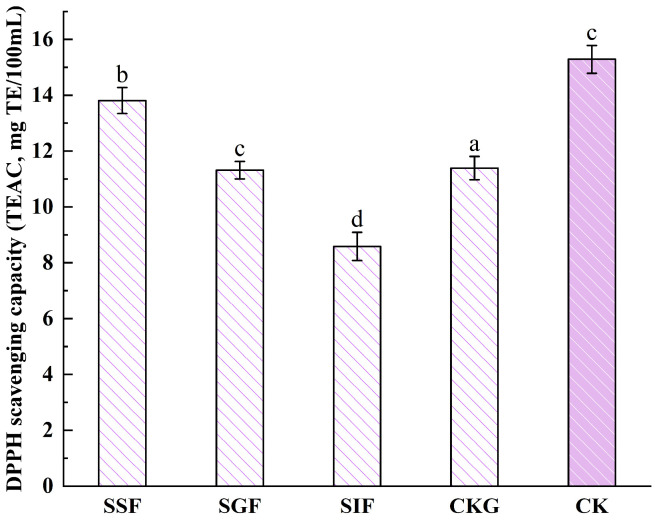
Changes in the DPPH radical-scavenging capacity of sugarcane vinegar during in vitro digestion. Different lowercase letters indicate statistically significant differences (*p* < 0.05).

Sugarcane vinegar exhibits the strongest DPPH free radical scavenging activity before digestion, with activity decreasing sequentially after oral, gastric, and intestinal digestion. This trend differs significantly from the changes in total phenol content, particularly evident during gastric and intestinal digestion. As previously noted, there were no significant differences in total phenol content between gastric and intestinal digestive fluids and the gastric acid control group, with bioaccessibility remaining above 90%. However, the DPPH scavenging ability of intestinal digestive fluids was 8.58 mg TE/100 mL, which was significantly lower than other groups. This result finding indicates that the relationship between antioxidant activity and total phenol content is not a simple linear one; physicochemical factors in the digestive environment play a crucial regulatory role in antioxidant function (Figure 1).

Different polyphenols have different sensitivities to the digestive environment. Some studies have shown that weakly alkaline conditions can trigger the enzymatic conversion of polyphenols, thereby increasing total phenol content and antioxidant activity [14]. Alkaline intestinal fluid (pH 7.5) can induce irreversible ring-opening, rearrangement, or polymerization reactions of flavonoids [15]. Although the degradation products are still detected as total phenols by the Folin–Ciocalteu method, they have lost the key ortho-diphenol active sites [30]. In addition, pepsin can protect polyphenols and flavonoids from excessive degradation to a certain extent [12,16], which is consistent with this study’s results, which shows that the DPPH scavenging capacity is stronger during gastric digestion than gastrointestinal. Based on the above analysis, the sharp drop in the DPPH scavenging capacity of sugarcane vinegar in the intestinal digestion stage is mainly attributed to the irreversible structural damage of flavonoids under the neutral pH conditions (pH 7.0) of the intestinal environment, where the B-ring ortho-dihydroxy structure and the C-ring are susceptible to oxidative degradation. Further qualitative and quantitative analyses of polyphenol components at different digestion stages should be conducted using LC-MS/MS.

#### 3.2.2. ABTS Scavenging Capacity

**Figure 2:**
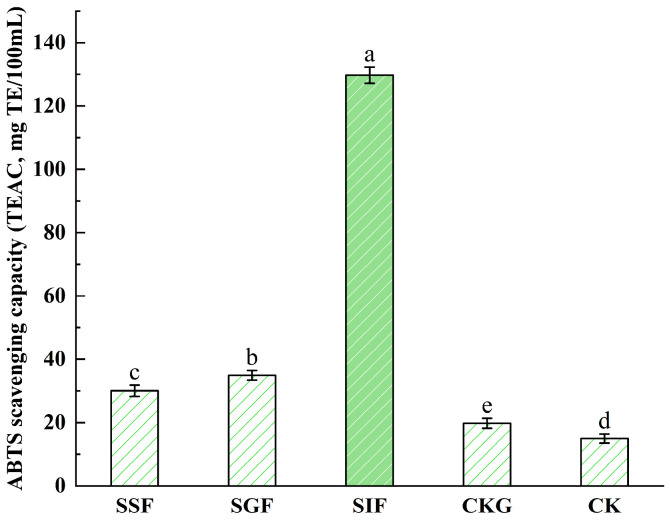
The variation in the ABTS radical scavenging capacity of sugarcane vinegar during in-vitro digestion. Different lowercase letters indicate significant differences (*p* < 0.05).

ABTS free radical scavenging capacity of the digestive fluid at each stage of the simulated digestion process for sugarcane vinegar was higher than control group, especially in the intestinal digestion stage (Figure 2). Zhao et al. [31] measured changes in the antioxidant capacity of perilla leaves and their extracts before and after simulated in vitro digestion. The results showed that the ability to scavenge ABTS free radicals was significantly improved after digestion, similar to those of this study. In addition to being affected by the content of active ingredients, antioxidant capacity is also affected by the pH and the type of active ingredients. It may be that the alkaline environment enhances the ability of some active ingredients to scavenge ABTS free radicals or promotes the synergistic ability of active ingredient monomers to scavenge ABTS free radicals. It may also the acid hydrolysis and alkaline hydrolysis can significantly improve the ABTS free radical scavenging capacity of bound phenols, and alkaline hydrolysis (small intestine) > acid hydrolysis (stomach) [28].

Zengin et al. [32] also found in their study of mulberry extract, that total phenol content increased after intestinal digestion (the Folin–Ciocalteu method still responded to degradation products), but the scavenging capacities of ABTS and DPPH continued to decline. This indicates that it is unreliable to evaluate antioxidant activity solely based on the total phenol content. Zhang et al. [17] further reported that about 46% of the metabolites in fermented chrysanthemum rice wine were positively correlated with decreases in DPPH, FRAP, and ORAC. In contrast, about 54% were positively correlated with increases in ABTS and HRSA. DPPH is a neutral free radical that is mainly cleared by hydrogen atom transfer (HAT). ABTS is a cationic free radical, which involves HAT and single electron transfer (SET). At the neutral pH (7.0) of the intestinal fluid, partial deprotonation of phenolic hydroxyl groups may occur, potentially enhancing single electron transfer capacity. This may be one of the reasons why the ABTS scavenging capacity does not decrease but increases during intestinal digestion, and the single electron transfer capacity is enhanced.

#### 3.2.3. Total Reducing Power

**Figure 3:**
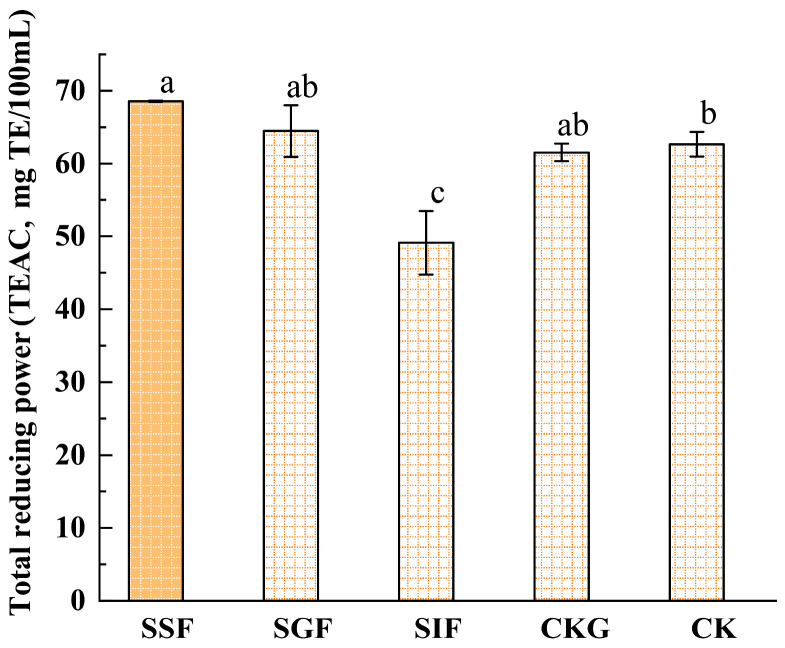
Changes in the total reducing power (TRP) of sugarcane vinegar during in vitro digestion. Different lowercase letters indicate significant differences (*p* < 0.05).

The total reducing power of sugarcane vinegar decreased significantly after gastrointestinal digestion. In contrast, the differences among the other four groups were not significant (Figure 3). The total flavonoid content and total reducing power of sugarcane vinegar showed similar trends during in vitro simulated digestion (Table 3). Therefore, it is believed that flavonoids are among the main components contributing to the reduction in the content of sugarcane vinegar during in vitro simulated digestion. The results of the antioxidant study of rose vinegar by Ozdemir et al. [10] showed that the total phenols and flavonoids of sugarcane are closely associated with its antioxidant capacity, and its reducing content decreases with the loss of flavonoid concentration. The synchronous changes of the total reducing power and total flavonoid content of sugarcane vinegar are consistent with the results of the present study. Sanarat et al. [33] also found, in their antioxidant study of sugarcane bagasse extract, that the contents of polyphenols, flavonoids, and tannins were positively correlated with the iron-reducing capacity (FRAP).

### 3.3. Ethanol Retention Rate of Sugarcane Vinegar

**Figure 4:**
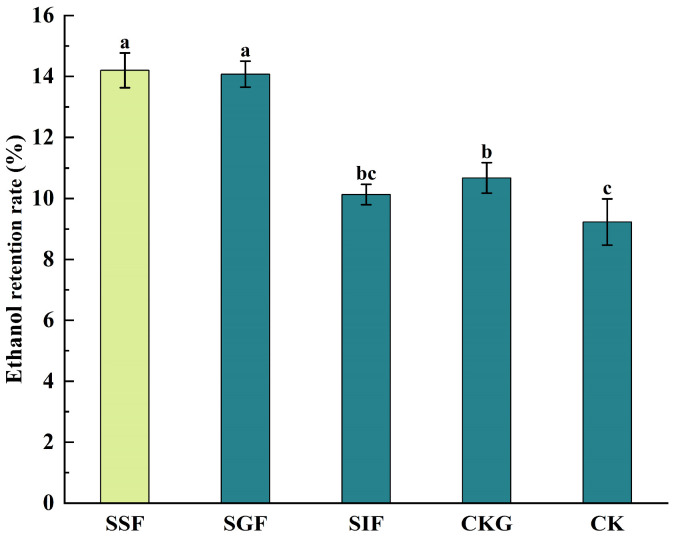
Changes in the ethanol retention rate (ERR) of sugarcane vinegar during in vitro digestion. Different lowercase letters indicate significant differences (*p* < 0.05).

Ethanol is generally absorbed into various tissues and organs of the body through oral cavity, esophagus, and gastrointestinal mucosa, and appears in the blood after about 5 min. By retaining ethanol with alcohol-degrading substances, the absorption of ethanol by the body can be reduced or delayed. Ethanol retention rate refers to the amount of free ethanol reduced by alcohol-degrading substances, thereby inhibiting or delaying the absorption of ethanol in the body [2]. As shown in Figure 4, the ethanol retention rate of sugarcane vinegar in the oral digestion stage (14.2%) and gastric digestion stage (14.1%) was significantly higher than before digestion (9.2%), indicating that sugarcane vinegar can effectively bind ethanol in the early stage of digestion and inhibit its absorption to a certain extent. However, during gastrointestinal digestion (10.1%), the ethanol retention rate decreased to a level not significantly different from that before digestion (*p* > 0.05), indicating that the binding effect was weakened or disappeared in the intestinal environment.

The similar studies have shown that the polyphenols and ethanol can form hydrogen-bonded complexes [34], while organic acids can form gels under acidic conditions, thereby slowing gastric emptying [35]. In addition, acidic polysaccharides can form gel networks in the stomach’s acidic environment, physically encapsulating ethanol [36]. However, under alkaline conditions in the intestine, polyphenols lose their hydrogen bond donor capacity due to deprotonation [37], pancreatic enzymes can hydrolyze polysaccharides, leading to the disintegration of the gel network [38], and bile salts can destroy the complex structure through interaction with polyphenols [39], ultimately leading to irreversible failure of the binding and release of ethanol.

This study is the first report of dynamic changes in ethanol retention rate in sugarcane vinegar during simulated in vitro digestion, approximately 14% during gastric digestion, and almost none during intestinal digestion. These results suggest that sugarcane vinegar is suitable for consumption before or at the beginning of alcohol consumption, as it can delay rather than completely prevent ethanol absorption.

### 3.4. Correlation Analysis

**Figure 5:**
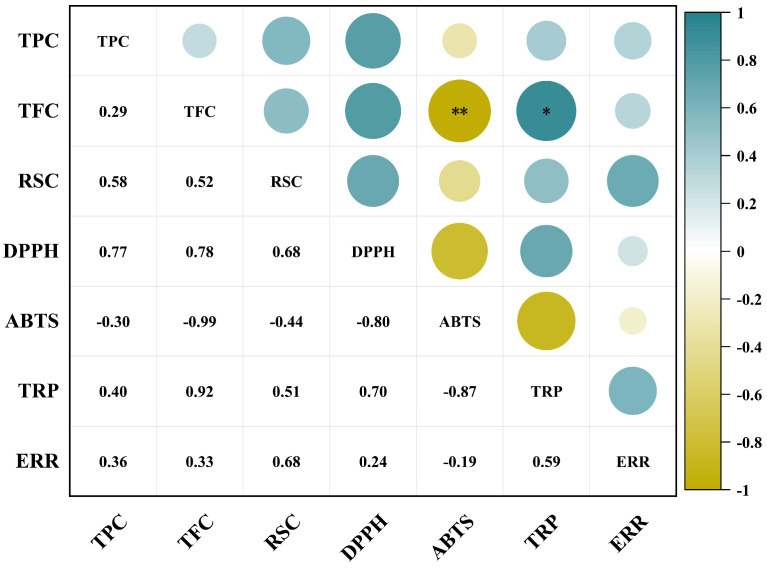
Analysis of the correlation between TPC, TFC, and RSC and antioxidant capacity and alcohol-metabolizing capacity. * and ** indicate statistical significance at *p*-value < 0.05 and <0.01. TPC: total phenolic content; TFC: total flavonoid content; RSC: reducing sugar content; TRP: total reducing power; ERR: ethanol retention rate.

The relationships among the content of active components in sugarcane vinegar, antioxidant capacity, and ethanol retention rate during in vitro digestion were further explored using Pearson correlation analysis (PCA). The total phenols, total flavonoids, and reducing sugars are all positively correlated with DPPH free radical scavenging capacity, total reducing capacity, and ethanol retention rate. Among them, the positive correlation between total flavonoids and total reducing capacity was significant. Total phenols, total flavonoids, and reducing sugars are negatively correlated with ABTS free radical scavenging capacity. The negative correlation between total flavonoids and ABTS free radical scavenging capacity was significant (Figure 5).

The contribution of different polyphenols to ABTS varies significantly. In some stages, non-flavonoids, such as phenolic acids and tannins, are stronger than flavonoids [13]. In this study, total flavonoids were significantly negatively correlated with ABTS scavenging capacity. Similar phenomena have been reported. This result can be due to the degradation of non-flavonoid polyphenols leading to a decrease in their contribution to ABTS, and the passive increase in the relative proportion of flavonoids; or the structural changes of flavonoids in the alkaline intestine, but still being detected by Folin–Ciocalteu [32], and the actual ABTS efficiency has been reduced. In contrast to ABTS, total flavonoids showed a significant positive correlation with iron-reducing content, consistent with the research findings of Sanarat et al. [33]. Total flavonoids in sugarcane bagasse extract showed positive correlation with iron-reducing power (*p* < 0.01). The potassium ferricyanide reduction method is based on electron transfer and sensitive to flavonoids that retain their full reducing capacity. This result confirms that flavonoids are the main contributor to the total reducing power of sugarcane vinegar; it also indirectly shows the negative correlation between total flavonoids and ABTS because flavonoids do not contribute to ABTS, but rather due to the degradation of non-flavonoid polyphenols or the low response of the ABTS method to flavonoid degradation products.

### 3.5. Effect of In Vitro Digestion on Polyphenolic Compounds in Sugarcane Vinegar

UPLC-MS was used to determine the polyphenolic content of sugarcane vinegar at different digestion stages (Table 3). In the undigested sample, ferulic acid had the highest content (2.781 ± 5.11 × 10^−2^ mg/L), followed by p-coumaric acid (2.219 ± 2.78 × 10^−2^ mg/L). Their changes during in vitro digestion were similar. There was no significant difference between oral digestion stage and the blank control. However, they increased significantly in the undigested stage and decreased significantly in the intestinal digestion stage and the gastric acid control group. Ferulic acid and p-coumaric acid are both cinnamic acid derivatives. Cinnamic acid and its derivatives in sugarcane vinegar may undergo hydroxylation, methylation, hydrolysis, and other reactions under the influence of enzymes or pH, thereby generating ferulic acid and p-coumaric acid [5]. Luteolin and salicylic acid showed the best stability during in vitro digestion, and there was no significant difference in their contents across the different stages.

**Figure 6:**
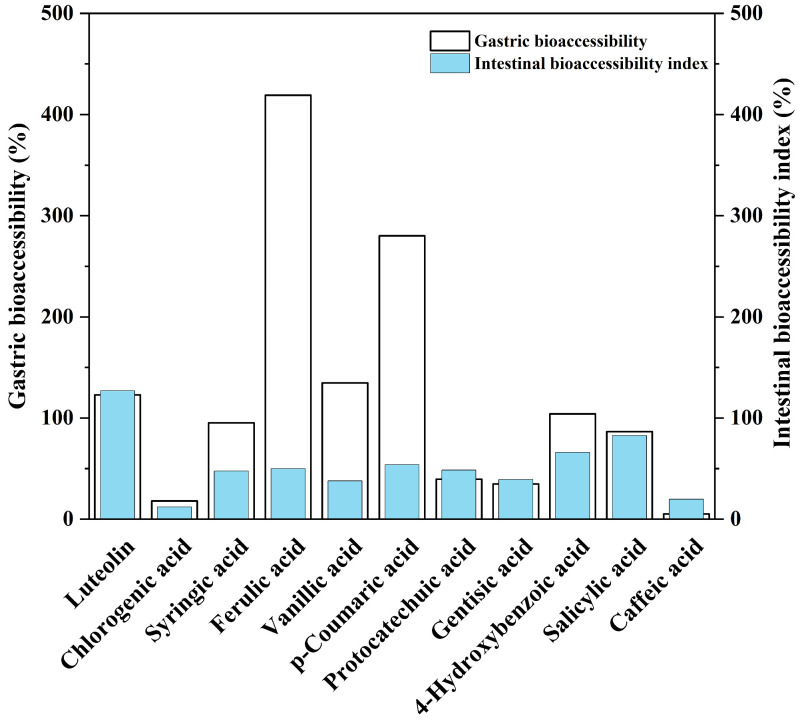
Bioaccessibility of sugarcane acetophenones during in vitro digestion.

From the perspective of bioaccessibility, the gastric digestion bioaccessibility of most sugarcane vinegar polyphenols is higher than gastrointestinal digestion (Figure 6). Ferulic acid has the highest gastric digestion bioaccessibility (419.10%), indicating that it is most easily bioavailable in the gastric phase; luteolin (125.00%) has the highest gastrointestinal digestion bioaccessibility, indicating that it is most easily bioavailable in the intestinal phase. Sęczyk et al. [18] found that the bioaccessibility ranges of ferulic acid and p-coumaric acid in different food matrices were 51–135%, and 131–173%, respectively. More than 100% bioaccessibility indicates that, after the bound phenolic acid undergoes enzymatic or chemical hydrolysis during digestion, the release amount exceeds the initial free amount. During gastric digestion, the synergistic action of gastric acid and pepsin promotes the efficient hydrolysis of the polysaccharide–phenolic acid complex in sugarcane vinegar, converting a large amount of bound ferulic acid into free form, thereby exhibiting extremely high bioaccessibility. Luteolin showed no significant difference in content across the different digestion stages, indicating that it was most stable in the in vitro digestion system. Cánovas et al. [40] conducted a systematic assessment of the bioaccessibility of olive leaf polyphenols in vitro and found that oleuropein and verbascoside were relatively stable in the gastric digestion stage but degraded significantly in the intestinal digestion stage, while luteolin-7-O-glucoside was the most stable during digestion (bioaccessibility was 43%).

In previous studies [1], the research group demonstrated that the ferulic acid, luteolin, and caffeic acids are the main active antioxidant components of sugarcane vinegar polyphenols. Among them, ferulic acid and luteolin exhibit strong synergistic antioxidant effects, consistent with the results of this study [5]. This study systematically revealed the changes in content and bioaccessibility of the main polyphenols in sugarcane vinegar during in vitro digestion, confirming that ferulic acid exhibits extremely high release efficiency during gastric digestion, and that luteolin maintains stable bioaccessibility throughout the digestion process.

The adsorption rate of sugarcane vinegar during oral and gastric digestion is about 14%, which is significantly higher than before digestion. At the same time, there is no significant difference between the intestinal digestion stage and pre-digestion stage. Based on this, it is hypothesized that sugarcane vinegar polyphenols alleviate alcoholic liver injury through different pathways, i.e., physical pathway shows the polyphenols inhibit ethanol absorption in the upper digestive tract by delaying gastric emptying through hydrogen bonds and organic acids, and by encapsulating ethanol in acidic polysaccharide gels, and biochemical pathway indicates the optimum bioaccessibility of phenolic substances, such as ferulic acid endows them with antioxidant stress resistance during digestion. In summary, sugarcane vinegar prevents alcoholic liver injury through a dual mechanism, such as physical inhibition of absorption and antioxidant regulation, in which polyphenols play a crucial role.

### 3.6. Network Pharmacology

#### 3.6.1. Potential Molecular Targets of Active Antioxidants and Compound Network Analysis

**Figure 7:**
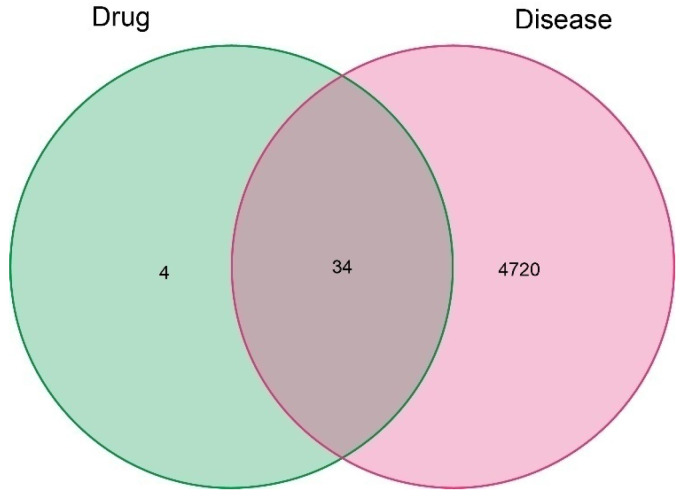
Venn diagram of sugarcane vinegar polyphenols and alleviated alcoholic liver injury targets. Drug represents sugarcane vinegar polyphenols, and disease represent targets.

The targets of 11 polyphenols from sugarcane vinegar were retrieved from the TCMSP database, yielding 38 target molecules. Then, targets related to alcoholic liver injury were identified using the GeneCards, OMIM, PharmGKB, TTD, and DrugBank databases. The union of the search results from these five databases yielded 4754 target molecules. Venn diagram analysis of the target molecules of sugarcane vinegar polyphenols and those related to alcoholic liver injury revealed 34 overlapping targets (Figure 7). These target molecules represent potential targets for sugarcane vinegar polyphenols to exert their anti-alcoholic liver injury effects.

**Figure 8:**
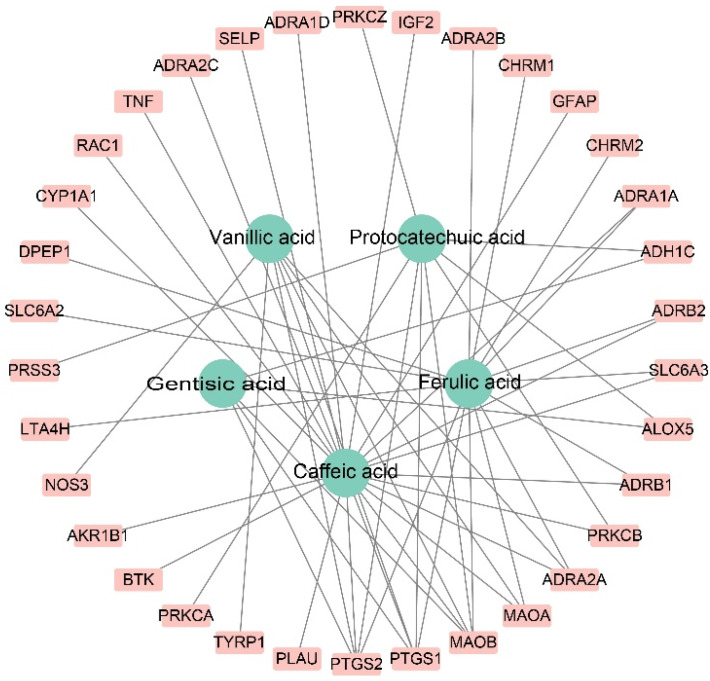
Network diagram of the active anti alcoholic liver injury components of sugarcane vinegar and their target genes for combating and reducing alcoholic liver disease. Antioxidants are represented by circles, while target genes are represented by rectangles.

A network diagram of sugarcane vinegar polyphenol compounds—targets of alcoholic liver injury was constructed in Cytoscape, and topological analysis was performed (Figure 8). The analysis results showed that five sugarcane vinegar polyphenols, such as ferulic acid, caffeic acid, protocatechuic acid, vanillic acid, and gentisic acid, act on alcoholic liver injury. The more edges a node has, the more targets it targets, and the greater the likelihood that it is a core component. The number of edges for the five sugarcane vinegar polyphenols, from largest to smallest, is caffeic acid > ferulic acid > protocatechuic acid > vanillic acid > gentisic acid. Similarly, the nodes of Monoamine Oxidase B (MAOB), Prostaglandin Endoperoxide Synthase 1 (PTGS1), and Prostaglandin Endoperoxide Synthase 2 (PTGS2) have the most edges (*n* = 5), indicating that they may be key targets of sugarcane vinegar polyphenols in combating alcoholic liver injury.

In the complex pathological process of alcoholic liver injury (ALD), Prostaglandin Endoperoxide Synthase 2 (PTGS2), Prostaglandin Endoperoxide Synthase 1 (PTGS1), and Monoamine Oxidase B (MAOB) play different but interconnected roles. Among them, PTGS2 is a key enzyme driving inflammation and oxidative stress in ALD. Its expression level is low in normal liver, but alcohol exposure can strongly induce its high expression in hepatocytes and Kupffer cells [1]. Related studies have shown that the buckwheat vinegar can significantly inhibit the expression of alcohol-induced PTGS2, ALOX5, and TRPM8, thereby improving alcoholic acute liver injury [6]. In alcoholic liver disease, MAOB can generate reactive oxygen species (ROS) through different pathways, exacerbating oxidative stress and liver damage, and further inducing PTGS2 expression [41,42]. PTGS1 primarily maintains liver homeostasis, while MAOB indirectly activates the PTGS2 pathway by generating ROS. The three interact to affect the progression of ALD jointly.

#### 3.6.2. Characteristic Analyses of Protein Pathway Network for Anti-Alcoholic Liver Injury

**Figure 9:**
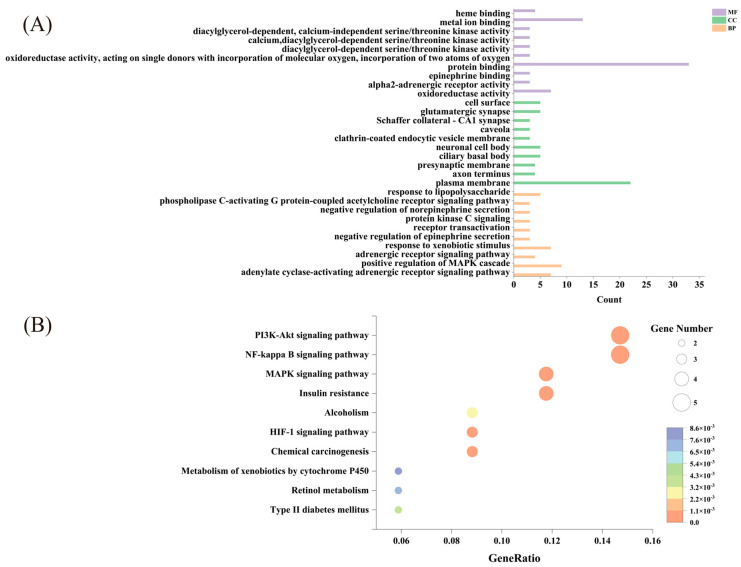
GO and KEGG enrichment analysis of sugarcane vinegar polyphenols to combat alcoholic liver injury. Panel (**A**) presents a bar graph of the top 10 GO terms across different categories, such as, biological process (BP), cellular compound (CC), and molecular function (MF). Panel (**B**) displays a bubble diagram of the top 10 enriched KEGG pathways.

Gene Ontology (GO) functional enrichment analysis was performed on 34 intersection targets of sugarcane vinegar polyphenols and alcoholic liver injury (Figure 9A). The results showed that 86 items were associated with biological processes (BP), 21 items with cellular components (CC), and 31 items with molecular functions (MF). The top 10 items closely related to alcoholic liver injury in each category (BP, CC, and MF) were selected for visualization (Figure 9A). Biological processes (BPs) mainly include the adenylate cyclase-activating adrenergic receptor signaling pathway, positive regulation of the MAPK cascade, adrenergic receptor signaling pathway, response to xenobiotic stimulus, and negative regulation of epinephrine secretion. In response to cellular components (CC), important entries include the plasma membrane, axon terminus, presynaptic membrane, ciliary basal body, and neuronal cell body. Molecular function (MF) entries are primarily enriched for oxidoreductase activity, alpha2-adrenergic receptor activity, epinephrine binding activity, protein binding activity, and oxidoreductase activity (acting on single donors with the incorporation of molecular oxygen, incorporating two oxygen atoms). Adrenergic signaling plays a significant role in alcoholic liver injury.

Previous research demonstrations have found that alcohol binge drinking may lead to hepatic steatosis and liver damage by activating lipolysis mediated by the sympathetic nerve-ADRB3 (β3-adrenergic receptor) in adipose tissue, while eliminating sympathetic nerve signals in adipose tissue or specifically knocking out ADRB3 in adipocytes can significantly reduce alcohol-induced hepatic lipid accumulation and inflammatory response [43,44]. Oxidative stress is a core driver of alcoholic liver injury. The activity of monooxygenases, such as CYP2E1 in the liver of patients with ALD is significantly increased, leading to excessive production of reactive oxygen species [3,45]. Reducing ROS production by regulating oxidoreductase activity can effectively mitigate ALD [3]. Previous studies have confirmed that sugarcane molasses polyphenol extract can reduce alcoholic liver injury by inhibiting CYP2E1 overexpression and regulating the Keap1/NF-κB signaling pathway, thereby exerting synergistic antioxidant and anti-inflammatory effects [5]. These findings effectively demonstrate that sugarcane molasses polyphenols reduce alcoholic liver injury by regulating biological processes in different cellular regions.

Kyoto Encyclopedia of Genes and Genomes (KEGG) pathway enrichment analysis showed the intersection targets of sugarcane vinegar polyphenols and alcoholic liver injury were significantly enriched in 118 pathways (*p* < 0.05) (Figure 9B). These pathways mainly involve NF-κB signaling, insulin resistance, PI3K-Akt signaling, HIF-1 signaling, alcoholism, and related pathways. Notably, the alcoholism signaling pathway is closely related to the persistence and relapse of alcohol addiction, involving the enrichment of genes of key enzymes in alcohol metabolism, such as ADH and ALDH. Studies have found that sugarcane polyphenols can significantly activate alcohol dehydrogenase and acetaldehyde dehydrogenase, supporting the main pathways of alcohol metabolism [4]. Another research demonstration also confirmed that sugarcane molasses polyphenol extract effectively alleviates alcohol-induced oxidative stress and inflammatory damage by inhibiting CYP2E1 overexpression, regulating the Keap1 signaling pathway, and inhibiting NF-κB pathway activation [5]. In addition, naturally occurring polyphenols are effective activators of ADH [7]. The above results indicate that sugarcane vinegar polyphenols can regulate alcohol metabolism-related pathways through multi-target, multi-pathway synergistic effects.

**Figure 10:**
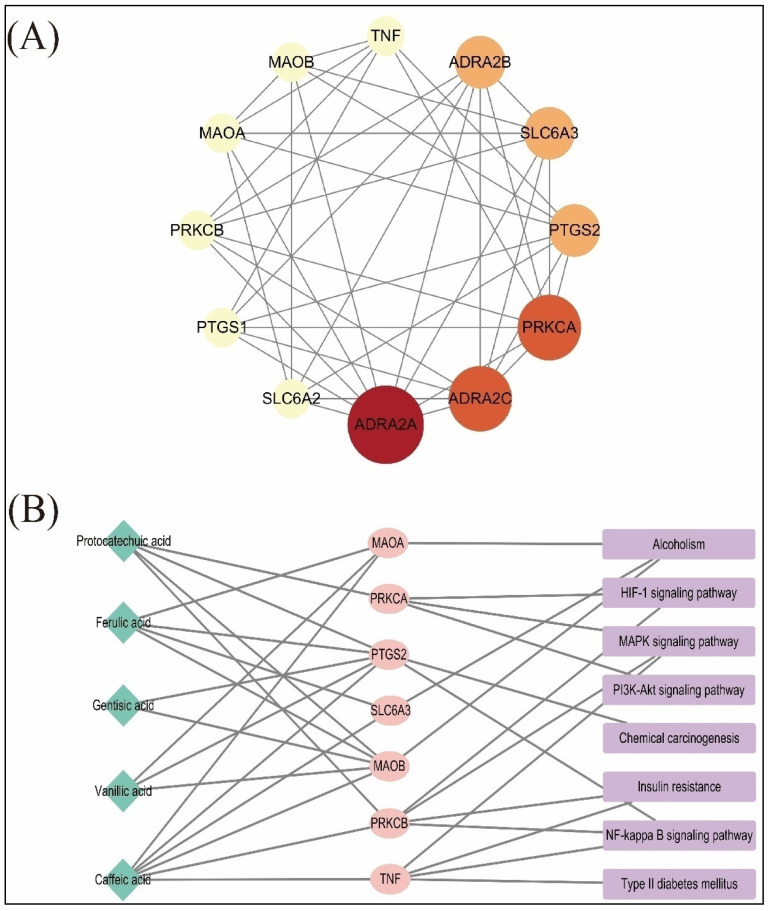
Protein–Protein interaction (PPI) network analysis of core targets screened from potential targets (**A**) and network diagram of the active component–key target interaction pathway (**B**) of sugarcane vinegar polyphenols to mitigate alcoholic liver injury. In Panel A, nodes are colored from light yellow to dark red and scaled by size according to their degree values, with darker red and larger nodes indicating higher degree centrality. Adrenoceptor Alpha 2A—ADRA2A; Adrenoceptor Alpha 2C—ADRA2C; Protein Kinase C Alpha—PRKCA; Prostaglandin-Endoperoxide Synthase 2—PTGS2; Solute Carrier Family 6 Member 3—SLC6A3; Adrenoceptor Alpha 2B—ADRA2B; Tumor Necrosis Factor—TNF; Monoamine Oxidase B—MAOB; Monoamine Oxidase A—MAOA; Protein Kinase C Beta—PRKCB; Prostaglandin Endoperoxide Synthase 1—PTGS1; Solute Carrier Family 6 Member 2—SLC6A2.

Based on the topological parameters, 12 core targets of sugarcane vinegar polyphenols in the treatment of alcoholic liver injury were identified, such as ADRA2A, ADRA2C, PRKCA, PTGS2, SLC6A3, ADRA2B, TNF, MAOB, MAOA, PRKCB, PTGS1, and SLC6A2 (Figure 10A). Seven of these targets were enriched in the KEGG signaling pathway associated with alcoholic liver injury. Arranged in descending order of core target enrichment, the signaling pathways are as follows Alcoholism = MAPK signaling pathway = NF-κB signaling pathway > HIF-1 signaling pathway = Insulin resistance > PI3K-Akt signaling pathway = Type II diabetes mellitus (Chemical carcinogenesis). The results in Figure 10B indicated that ferulic acid, vanillic acid, and caffeic acid can work together to target MAOA and MAOB. Previous studies have shown that the ferulic acid can significantly inhibit MAO-A activity, and caffeic acid derivatives have a strong inhibitory effect on MAO-B [8]. Meanwhile, MAOA has been confirmed as a key regulatory target for alleviating alcoholic liver injury [46]. At the same time, MAOB plays an important role in ethanol-induced cell damage, and MAOB inhibitors can block the ethanol-induced damage cascade [9]. Based on the above findings, it is speculated that ferulic acid, vanillic acid, and caffeic acid can act synergistically to target MAOA and MAOB and regulate the alcohol poisoning signaling pathway, which may be one of the key mechanisms by which cane vinegar alleviates alcoholic liver injury.

#### 3.6.3. Molecular Docking of Active Anti Alcoholic Liver Disease Components and Their Key Targets

**Figure 11:**
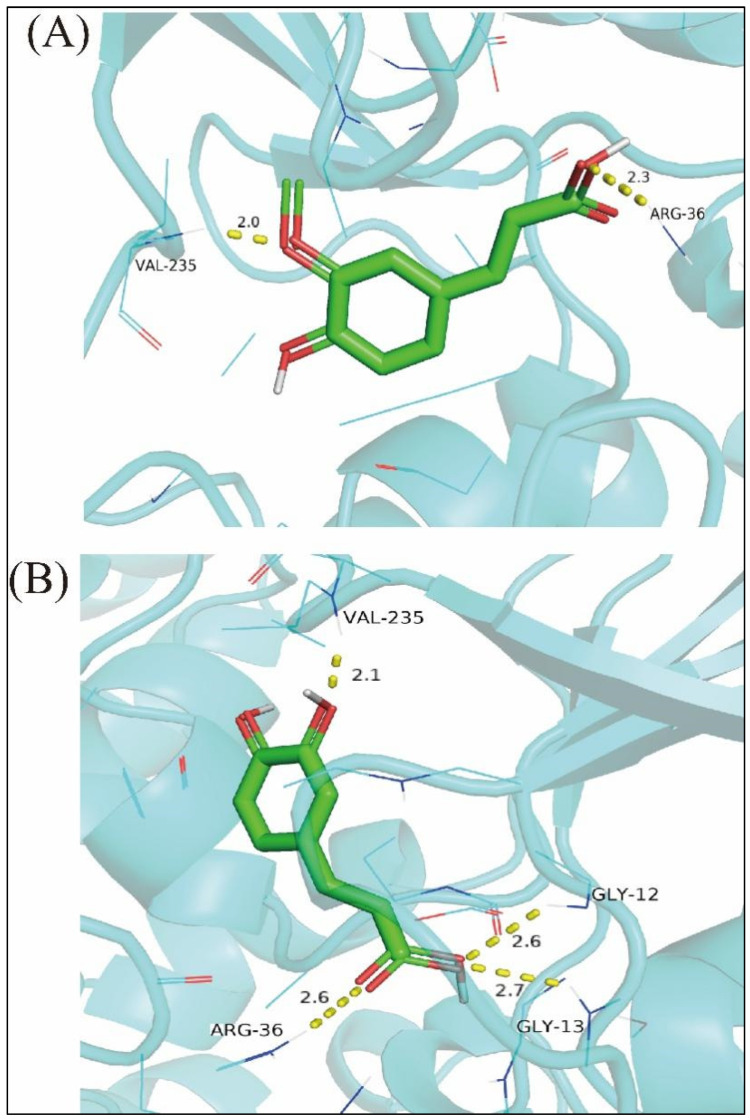
Molecular docking model diagrams of ferulic acid with MAOB (**A**), and caffeic acid with MAOB (**B**). Hydrogen bonds are shown as green dashed lines with distances (Å), and hydrophobic interactions are shown as red arcs. The interacting amino acid residues (e.g., ARG-36, VAL-235, GLY-12, GLY-13) are labeled. Docking was performed using AutoDock, and the interaction diagrams were visualized using PyMOL 2.3.2.

Molecular docking can effectively analyze molecular interactions. Molecular docking was performed on five sugarcane vinegar polyphenols, such as ferulic acid, caffeic acid, protocatechuic acid, vanillic acid, and gentisic acid, and seven core targets, i.e., MAOA, PRKCA, PTGS2, SLC6A3, MAOB, PRKCB, and TNF (Table 4). The minimum binding energy is in the range of −7.9 kcal·mol^−1^ to −5.7 kcal·mol^−1^, and the binding free energy is less than −5.0 kcal/mol, indicating that the compound and the target have good binding activity [20]. The hydrogen bond distance is in the range of 1.9 to 2.8 Å, which is smaller than conventional hydrogen bond (3.5 Å), indicating that the binding conformation is stable [21]. Among the various amino acids, ferulic acid exhibited the lowest binding energy (−7.9 kcal·mol^−1^) to MAOB, with potential binding sites at amino acid residues VAL-235 and ARG-36, with hydrogen bond distances of 2.0 Å and 2.3 Å, respectively. Caffeic acid (MAOB) followed, with a binding energy of −7.7 kcal·mol^−1^ and potential binding sites at amino acid residues VAL-235, GLY-12, GLY-13, and ARG-36, with hydrogen bond distances of 2.1, 2.6, 2.7, and 2.6 Å, respectively. This suggests that their binding conformation is the most stable (Figure 11). Ferulic acid and caffeic acid can bind to the same target sites at the same locations, such as VAL-235 and ARG-36 of MAOB. This further illustrates that cane vinegar can exert its anti-alcoholic liver injury effect through the combined action of ferulic acid and caffeic acid on the Alcoholism signaling pathway. Nevertheless, no studies have been found to explore the synergistic anti-alcoholic liver injury effects of ferulic acid and caffeic acid by modulating the Alcoholism signaling pathway, underscoring the need for further research.

## 4. Conclusions and Future Research Directions

The present research systematically assessed the effects of simulated in vitro gastrointestinal digestion on the active components, antioxidant activity, and ethanol inhibition capacity of sugarcane vinegar, and further elucidated its molecular mechanism against ALD using network pharmacology. Results showed that the total phenols (TPC), total flavonoids (TFC), reducing sugars (RSC), and polyphenol monomers exhibited significantly different trends during digestion. The bioaccessibility of TPC remained above 90% during gastric digestion, while TFC decreased significantly (39.39 ± 0.85%) after combined gastrointestinal digestion. Antioxidant activity assays revealed that the DPPH radical scavenging capacity and total reducing capacity of sugarcane vinegar decreased gradually during gastrointestinal digestion; conversely, the ABTS radical scavenging capacity reached its peak during this process. Alcohol excretion experiments showed that the ethanol retention rate of sugarcane vinegar during gastric digestion was approximately 14%, significantly higher than undigested sample. Correlation analysis confirmed that antioxidant activity was closely correlated with phenolic compound content. Further analysis of the changes in the content of phenolic monomers during digestion using UPLC-MS revealed that most polyphenols had higher gastric bioaccessibility than gastrointestinal bioaccessibility. Ferulic acid exhibited the highest gastric bioaccessibility index (419.10%), indicating the highest rate of release and utilization in the gastric phase; luteolin showed the highest gastrointestinal bioaccessibility (125.00%), demonstrating high bioaccessibility even in the intestinal tract. The observed digestion-phase bioaccessibility and antioxidant activity profiles mechanistically support the retained in vivo functional activity of sugarcane vinegar. Phenolic acids (especially ferulic acid) are efficiently released in the gastric phase, luteolin remains stable through the intestinal phase, and the bioaccessible fractions maintain appreciable antioxidant capacity despite digestion-induced compositional changes. Network pharmacology and molecular docking analyses further elucidated the molecular basis of cane vinegar’s anti-ALD effect. The core targets include ADRA2A, ADRA2C, PRKCA, PTGS2, SLC6A3, ADRA2B, TNF, MAOB, MAOA, PRKCB, PTGS1, and SLC6A2. The binding free energies of five key polyphenols, such as caffeic acid, ferulic acid, protocatechuic acid, vanillic acid, and gentisic acid, to these targets range from −7.9 to −5.7 kcal/mol, and hydrogen bonding interactions are present in all binding conformations, indicating a better binding affinity between the ligands and the targets. These compounds can regulate multiple signaling pathways, including Alcoholism, MAPK, NF-κB, and PI3K-Akt, synergistically exerting anti-ALD activity.

Sugarcane vinegar, a high-value-added product derived from sugarcane processing byproducts, has the potential to be developed into a functional food for preventing and treating ALD. This study is the first demonstration of changes in the bioaccessibility and alcohol-detoxifying ability of the active ingredients in sugarcane vinegar during in vitro digestion. It systematically reveals the potential mechanism by which its polyphenolic components combat ALD through a multi-target–multi-pathway network. The limitations of this study are the in vitro digestion model cannot fully simulate the dynamic process in vivo, and the specific degradation products generated during digestion were not identified. Future research could use the Caco-2 cell model to assess the actual bioaccessibility of the active ingredients and verify the key targets predicted by network pharmacology through animal experiments, providing a more comprehensive scientific basis for the development of functional foods based on the sugarcane vinegar.

## Figures and Tables

**Table 1:** The chemical composition of simulated gastrointestinal digestion juices.

Constituent	Stock Concentration		Simulated Salivary Fluid (SSF)		Simulated Gastric Fluid (SGF)		Simulated Intestinal Fluid (SIF)	
	g/L	mol/L	Stock Volume (mL)	Concentration (mmol/L)	Stock Volume (mL)	Concentration (mmol/L)	Stock Volume (mL)	Concentration (mmol/L)
KCl	37.3	0.5	15.1	15.1	6.9	6.9	6.8	6.8
KH_2_PO_4_	68	0.5	3.7	3.7	0.9	0.9	0.8	0.8
NaHCO_3_	84	1	6.8	13.6	12.5	25	42.5	85
NaCl	117	2	-	-	11.8	47.2	9.6	38.4
MgCl_2_(H_2_O_6_)	30.5	0.15	0.5	0.15	0.4	0.12	1.1	0.33
(NH_4_)_2_CO_3_	48	0.5	0.06	0.06	0.5	0.5	-	-
pH	-	-	7.0		3.0		7.0	

**Table 2:** Changes in the total phenols, flavonoids, and reducing sugars, and their bioaccessibility index during in vitro digestion of sugarcane vinegar.

Indicators	TPC (µg/mL)	TFC (µg/mL)	RSC (mg/mL)
SSF	605.74 ± 17.05 ^a^	260.57 ± 5.64 ^b^	1.71 ± 0.06 ^a^
SGF	553.13 ± 9.51 ^b^	264.39 ± 2.09 ^ab^	1.75 ± 0.07 ^a^
GBI (%)	92.94 ± 1.55	97.85 ± 2.69	99.03 ± 1.49
SIF	560.62 ± 10.91 ^b^	107.06 ± 1.84 ^c^	1.50 ± 0.03 ^b^
IBI (%)	96.07 ± 1.03	39.39 ± 0.85	84.88 ± 1.01
CKG	550.94 ± 6.77 ^b^	258.86 ± 2.49 ^b^	1.43 ± 0.02 ^b^
CK	595.90 ± 3.72 ^a^	269.79 ± 5.17 ^a^	1.77 ± 0.01 ^a^

Note: SSF—simulated salivary fluid; SGF—simulated gastric fluid; GBI—gastric bioaccessibility index; SIF—simulated intestinal fluid; IBI—intestinal bioaccessibility index; CKG—gastric acid control; CK—blank control (sugarcane vinegar diluted with distilled water to the same volume as the digested samples). Different letters represent statistically significant differences (*p* < 0.05).

**Table 3:** Changes in the content of individual phenols in sugarcane vinegar during in vitro digestion.

Phenolic Compounds	Phenolic Content Before and After In Vitro Gastrointestinal Digestion				
	SSF (mg/L)	SGF (mg/L)	SIF (mg/L)	CKG (mg/L)	CK (mg/L)
Luteolin	0.027 ± 5.49 × 10^−4^ ^a^	0.029 ± 2.01 × 10^−3 a^	0.030 ± 2.20 × 10^−3 a^	0.024 ± 8.04 × 10^−4 b^	0.024 ± 1.01 × 10^−3 b^
Chlorogenic acid	0.295 ± 7.38 × 10^−3 a^	0.048 ± 1.85 × 10^−3 d^	0.032 ± 0.00218 ^e^	0.143 ± 8.81 × 10^−3 c^	0.269 ± 1.08 × 10^−2 b^
Syringic acid	0.449 ± 4.01 × 10^−2 a^	0.479 ± 4.18 × 10^−2 a^	0.188 ± 1.04 × 10^−2 c^	0.308 ± 2.27 × 10^−3 b^	0.503 ± 2.48 × 10^−2 a^
Ferulic acid	2.797 ± 3.30 × 10^−2 b^	11.653 ± 3.35 × 10^−1 a^	1.391 ± 4.60 × 10^−2 d^	1.901 ± 1.02 × 10^−1 c^	2.781 ± 5.11 × 10^−2 b^
Vanillic acid	0.217 ± 7.29 × 10^−4 b^	0.315 ± 1.59 × 10^−2 a^	0.088 ± 4.58 × 10^−3 d^	0.136 ± 7.18 × 10^−3 c^	0.234 ± 8.31 × 10^−3 b^
*p*-Coumaric acid	2.157 ± 3.30 × 10^−2 b^	6.216 ± 0.00962 ^a^	1.197 ± 8.21 × 10^−2 c^	1.648 ± 6.50 × 10^−2 c^	2.219 ± 2.78 × 10^−2 b^
Protocatechuic acid	0.164 ± 1.95 × 10^−3 a^	0.068 ± 2.76 × 10^−3 d^	0.084 ± 4.85 × 10^−3 c^	0.123 ± 6.87 × 10^−3 b^	0.173 ± 9.14 × 10^−3 a^
Gentisic acid	0.188 ± 3.39 × 10^−3 b^	0.069 ± 2.237 × 10^−3 e^	0.078 ± 1.78 × 10^−3 d^	0.116 ± 2.96 × 10^−3 c^	0.199 ± 1.40 × 10^−3 a^
4-Hydroxybenzoic acid	0.063 ± 1.93 × 10^−3 b^	0.071 ± 3.44 × 10^−3 a^	0.045 ± 2.70 × 10^−3 c^	0.043 ± 2.40 × 10^−4 c^	0.068 ± 3.383 × 10^−3 ab^
Salicylic acid	0.05 ± 1.41 × 10^−3 b^	0.051 ± 7.37 × 10^−4 b^	0.048 ± 7.83 × 10^−4 b^	0.049 ± 2.42 × 10^−4 b^	0.058 ± 3.45 × 10^−3 a^
Caffeic acid	0.283 ± 1.49 × 10^−3 b^	0.015 ± 5.80 × 10^−4 e^	0.057 ± 4.64 × 10^−4 d^	0.198 ± 6.62 × 10^−4 c^	0.287 ± 1.71 × 10^−4 a^

Note: All compounds were quantified using authentic reference standards (purity ≥ 98%) by external standard calibration with UPLC-MS/MS. Different lowercase letters in the same row indicate significant differences (*p* < 0.05).

**Table 4:** Molecular docking simulation of bioactive compounds from sugarcane vinegar extract and their protein targets.

Molecule Name	Target	Residues Associated in H Bonding	H-Bond Length (Å)	Docking Energy (kcal/mol)
Caffeic acid	MAOA	ASN-179, LYS357	2.3, 2.1	−6
Caffeic acid	MAOB	VAL-235, GLY-12, GLY-13, ARG-36	2.1, 2.6, 2.7, 2.6	−7.7
Caffeic acid	PRKCB	ARG-142, GLN-68, PHE-501, ASN-30	2.2, 2.0, 2.1, 2.0, 2.0 2.2	−6.5
Caffeic acid	PTGS2	GLN-374, TYR-373, GLY-533, ASN-537	2.8, 2.1, 2.5, 2.0, 2.4	−6.9
Caffeic acid	SLC6A3	PHE-320	1.9	−7
Caffeic acid	TNF	LEU-85, HIS-110, ARG-76	2.1, 2.1, 2.2	−6.7
Ferulic acid	MAOA	GLY-443,	2.1	−6.9
Ferulic acid	MAOB	VAL-235, ARG-36	2.0, 2.3	−7.9
Ferulic acid	PTGS2	ARG-44, LYS-137, GLN-461	2.2, 2.3, 2.7, 2.8, 2.0 2.1, 2.1	−6.8
Ferulic acid	SLC6A3	ALA-81, LEU-80, SER-422	2.3, 2.7, 2.5	−6.7
Gentisic acid	MAOB	ALA-35, VAL-235, LEU-268	1.9, 2.5,2.5	−7.1
Gentisic acid	PTGS2	ASN-375, TYR-373, GLN-374	1.9, 2.3, 1.9, 2.6	−6.3
Protocatechuic acid	MAOB	ALA-35, VAL-235	2.2, 2.3	−7.1
Protocatechuic acid	PRKCA	TRP-512, PHE-452, LYS-456, GLY-587	2.4, 2.2, 2.1, 2.6	−6.3
Protocatechuic acid	PRKCB	THR-321, ILE-651, GLN-653,ARG-77	2.0, 2.2, 2.2, 2.3	−5.7
Protocatechuic acid	PTGS2	ASP-229, TRP-139, ARG-333,GLU-140, GLN-241, GLY-235	2.8, 2.2, 2.2, 2.3, 2.2, 2.5	−6.2
Vanillic acid	MAOA	SER-24, ILE-23, TYR-407	2.4, 2.2, 2.7	−6.6
Vanillic acid	MAOB	ALA-35, GLU-34	2.7, 2.4	−7
Vanillic acid	PTGS2	ASN-144, GLN-241, ARG-333,	2.7, 2.5, 2.1, 2.6, 2.1	−6.3

## Data Availability

The original contributions presented in this study are included in the article. Further inquiries can be directed to the corresponding authors.
